# MicroRNA-like RNA Functions Are Required for the Biosynthesis of Active Compounds in the Medicinal Fungus *Sanghuangporus vaninii*

**DOI:** 10.1128/spectrum.00219-22

**Published:** 2022-10-27

**Authors:** Qiumei Zhou, Xuebing Yin, Hanghang Zhang, Yulong Wang

**Affiliations:** a Experimental Center of Clinical Research, The First Affiliated Hospital of Anhui University of Chinese Medicine, Hefei, China; b Anhui Provincial Key Laboratory of Microbial Pest Control, Anhui Agricultural University, Hefei, China; c Nanling Forestry Technology Center, Nanling Forestry Bureau, Nanling, China; Weizmann Institute of Science

**Keywords:** *Sanghuangporus vaninii*, RNA-dependent RNA polymerase, Dicer, Argonaute, spray-induced gene silencing, microRNA-like RNA, bioactive compounds

## Abstract

miRNA-like RNAs (milRNAs) have been recognized as sequence-specific regulators of posttranscriptional regulation of gene expression in eukaryotes. However, the functions of hundreds of fungal milRNAs in the biosynthesis of metabolic components are obscure. *Sanghuangporus* produces diverse bioactive compounds and is widely used in Asian countries. Here, genes encoding two Dicers, four Argonautes, and four RdRPs were identified and characterized in *Sanghuangporus vanini*. Due to the lack of an efficient gene manipulation system, the efficacy of spray-induced gene silencing (SIGS) was determined in *S. vanini,* which showed efficient double-stranded RNA (dsRNA) uptake and gene silencing efficiency. SIGS-mediated gene knockdown showed that SVRDRP-3, SVRDRP-4, SVDICER-1, and SVDICER-2 were critical for mycelial biomass, flavonoid, triterpenoid, and polysaccharide production. Illumina deep sequencing was performed to characterize the milRNAs from *S. vanini* mycelium and fruiting body. A total of 31 milRNAs were identified, out of which, *SvmilR10*, *SvmilR17*, and *SvmilR33* were *Svrdrp-4*- and *Svdicer-1*-dependent milRNAs. Importantly, SIGS-mediated overexpression of *SvmilR10* and *SvmilR33* resulted in significant changes in the yields of flavonoids, triterpenoids, and polysaccharides. Further analysis showed that these milRNA target genes encoding the retrotransposon-derived protein PEG1 and histone-lysine N-methyltransferase were potentially downregulated in the milRNA overexpressing strain. Our results revealed that *S. vanini* has high external dsRNA and small RNA uptake efficiency and that milRNAs may play crucial regulatory roles in the biosynthesis of bioactive compounds.

**IMPORTANCE** Fungi can take up environmental RNA that can silence fungal genes with RNA interference, which prompts the development of SIGS. Efficient dsRNA and milRNA uptake in *S. vanini*, successful dsRNA-targeted gene block, and the increase in intracellular miRNA abundance showed that SIGS technology is an effective and powerful tool for the functional dissection of fungal genes and millRNAs. We found that the RdRP, Dicer, and Argonaute genes are critical for mycelial biomass and bioactive compound production. Our study also demonstrated that overexpressed SVRDRP-4- and SVDICER-1-dependent milRNAs (*SvmilR10* and *SvmilR33*) led to significant changes in the yields of the three active compounds. This study not only provides the first report on SIGS-based gene and milRNA function exploration, but also provides a theoretical platform for exploration of the functions of milRNAs involved in biosynthesis of metabolic compounds in fungi.

## INTRODUCTION

Macrofungi, such as *Ophiocordyceps sinensis*, *Polyporus umbellatus,* and Ganoderma lucidum, have been utilized as an important pool of bioresources due to their abundant and high diversity of bioactive compounds (1–4). Compared with these species, *Sanghuang* has received increasing attention for its medicinal functions, including antitumor, antioxidant, anti-inflammation, and immunomodulation activities (5, 6). Although *Sanghuang*, documented for more than 2,000 years, was confirmed to have significant antitumor properties in 1968, little is known about the fungal molecular mechanism due to the lack of methodologies to test gene function *in vivo* (5–7). In fact, few investigations of gene function have been reported from Hymenochaetales, although many of these fungal genomic sequencing analyses have been performed (6, 8–11).

RNA interference (RNAi), a conserved mechanism in eukaryotes, including fungi, mediates gene regulation and has emerged as a powerful tool to study gene function in a wide range of eukaryotic organisms (12, 13). Delivery of double-stranded RNA (dsRNA) with RNAi mediates RNA destruction and could reduce the expression of gene targets (14). Recent studies have discovered that most fungal species contain the RNAi machinery, and gene knockdown is achievable with the introduction of dsRNA molecules (15–17). The efficiency of dsRNA uptake is critical to the success of sequence-specific knockdown of gene targets and varies across fungal species. Some plant pathogens, such as Botrytis cinerea, Rhizoctonia solani, and Aspergillus niger, showed highly efficient dsRNA uptake; *Trichoderma virens* revealed weak uptake, while no uptake was observed in *Colletotrichum gloeosporioides* (16). Taken together, these data prompted the development of an innovative technology, spray-induced gene silencing (SIGS), for fungal gene research by knockdown of specific genes.

MicroRNAs (miRNAs), typically ranging from 18 to 24 nucleotides in length, may mediate gene silencing in fungi (18, 19). Recent research has revealed that miRNAs exist and play essential roles in the development of Basidiomycetes species (20). In *G. lucidum*, more than one hundred miRNAs were identified, and functional enrichment analysis showed that their target genes were involved in various physiological and cellular differentiation processes, such as the biosynthesis of triterpenes and polysaccharides and the lignin degradation pathway (21, 22). A total of 22 and 7 miRNAs were found during Coprinopsis cinerea development (vegetative mycelium, primordium and basidiospore germination), and some miRNAs, cci-milR-37, cci-milR-12c, and cci-milR-13e-5p, were found to be potential regulators for fruiting body development and vegetative hyphal growth, including pheromones and hydrophobin (23, 24). High-throughput sequencing and qPCR analysis confirmed that some Ago1-mediated miRNAs could facilitate cryogenic autolysis progress with regulation of signal transduction and ubiquitination in *Volvariella volvacea* (25). These miRNA analyses help to further understand the regulation of fungal physiology and development; however, no miRNA, to date, has been reported at the genomic level in Hymenochaetales.

Recently, we reported genomic information for *Sanghuangporus vaninii*, which has been used for the large-scale industrial production of fruiting bodies in China. Genes and gene clusters involved in secondary metabolism and their regulation have been identified by transcriptome analysis (8, 26). Here, we identified miRNA-like RNAs (milRNAs) during different developmental stages with high-throughput sequencing and determined the potential functions of milRNAs in the fungal biosynthesis of bioactive compounds with SIGS. Overall, this is the first genome-wide characterization of the functions of milRNAs and milRNAs in bioactive compound biosynthesis in Hymenochaetales.

## RESULTS

### Phylogenetic and expression profile analyses of AGO, Dicer, and RdRP proteins.

**FIG 1:**
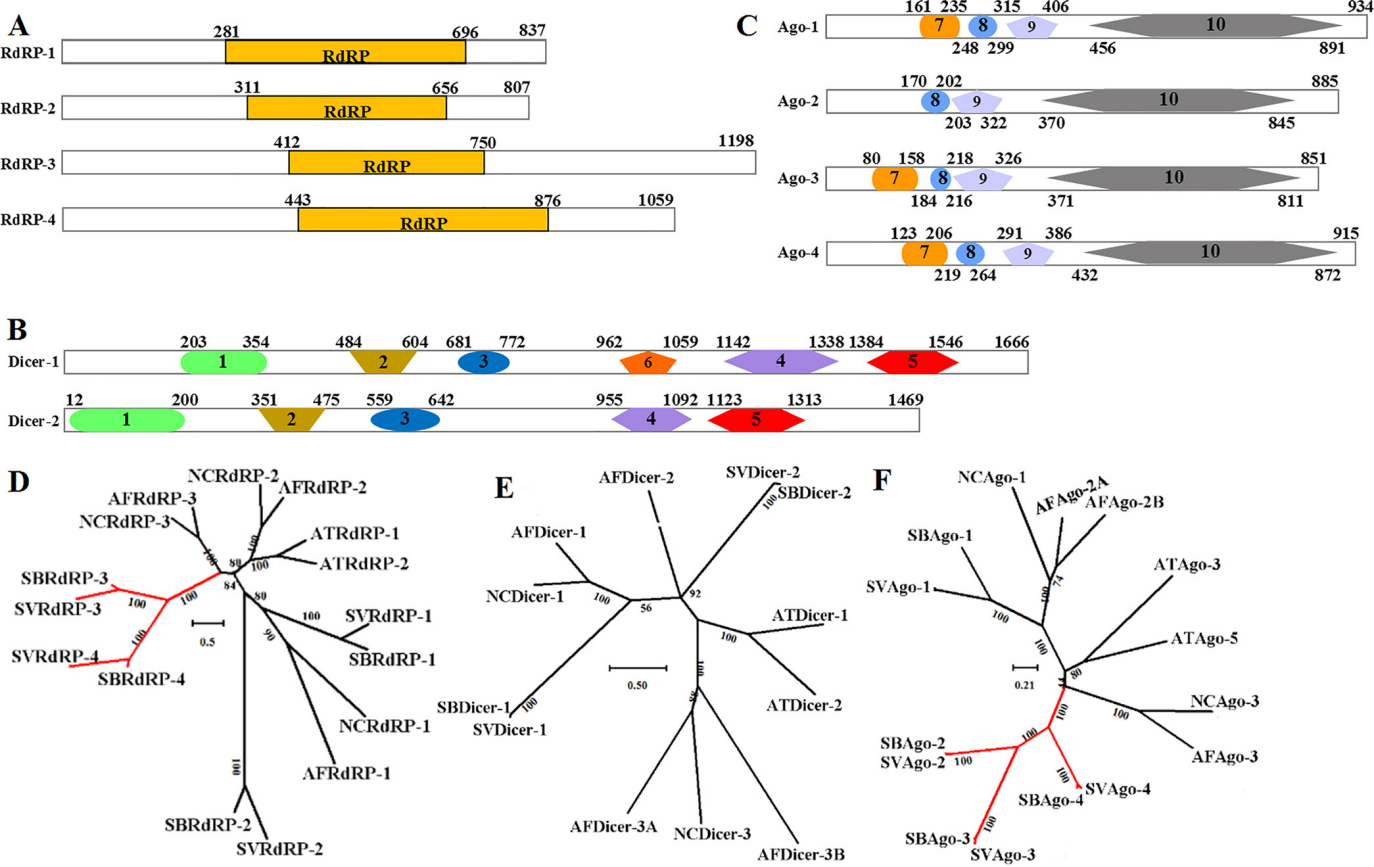
Domain structure and phylogeny of RdRPs, Dicers, and AGOs. Typical domains of RdRPs (A), Dicers (B), and AGOs (C) in *S. vaninii*. Phylogenetic analysis of RdRPs (D), Dicers (E), and AGOs (F) in *S. vaninii* (SV) and other fungi. RdRP, RdRP domain; 1, DEXHc_dicer domain; 2, helicase_C domain; 3, Dicer_dimer domain; 4 and 5, RNase III domains; 6, PAZ domain; 7, ArgoN domain; 8, ArgoL1 domain; 9, PAZ domain; 10, Piwi domain. NC, Neurospora crassa; AF, Aspergillus flavus; AT, Arabidopsis thaliana. SB, *Sanghuangporus baumii*. The red clade denotes proteins from *S*. *vaninii* and *S*. *baumii* that were resolved into distinct groups.

RNA-dependent RNA polymerase (RdRP), Argonaute (AGO), and Dicer proteins are key components of miRNA maturation and function in fungi, and possessing these proteins could be evidence for the presence of miRNAs in *S. vaninii*. The RdRP, AGO, and Dicer proteins of N. crassa were chosen as reference sequences for their intensive study (18). Four RdRPs, two Dicers, and four AGOs were identified using BLAST analysis in the genome of *S. vaninii* (Fig. 1). All four identified RdRP proteins contain the typical RdRP domain, whereas they differ in length by 807, 837, 1.059, and 1.198 amino acids, respectively (Fig. 1A). Both Dicer proteins contain five conserved domains, including a DEXHc_dicer domain, a helicase_C domain, a Dicer_dimer domain, and two RNase III domains; Dicer-2 also includes a PAZ domain (Fig. 1B). AGO-1, AGO-3, and AGO-4 contain four conserved domains, an ArgoN domain, an ArgoL1 domain, a PAZ domain, and a Piwi domain, whereas no ArgoN domain was searched in AGO-2 (Fig. 1C).

Phylogenetic analysis showed that the number of RdRPs varied among different fungi; *S*. *vaninii* and *S*. *baumii* possessed four RdRPs, while three RdRPs were detected in N. crassa and A. flavus (Fig. 1D). RdRP-3 and RdRP-4 in *Sanghuangporus* species formed supported monophyletic clades that are sister to each other, suggesting distinct roles among these proteins in different fungi. The number of Dicers varied from two (*S*. *vaninii* and *S*. *baumii*) to four (A. flavus) (Fig. 1E). In the Dicer-1 group, *S*. *vaninii* and *S*. *baumii* formed separate clades and shared 56% sequence similarity with N. crassa and A. flavus. The phylogenetic tree constructed on the basis of the AGO protein sequences showed that AGO-2, AGO-3, and AGO-4 from *S*. *vaninii* and *S*. *baumii* were resolved into a distinct group (Fig. 1F).

**FIG 2:**
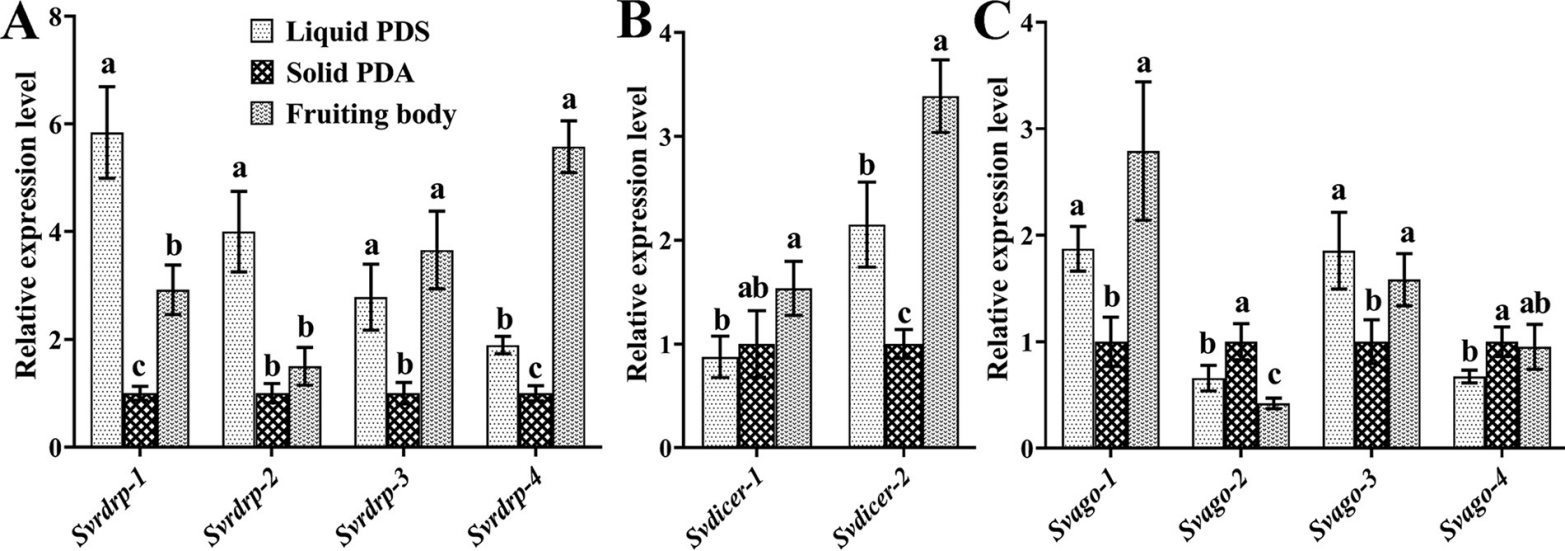
qPCR analysis of the RdRP, Dicer and AGO genes in *S. vaninii* cultured with different media. Samples collected from the solid PDA (20 days), liquid PDS (10 days), and fruiting bodies (3 years old) were used for qPCR analysis of RdRP (A), Dicer (B), and AGO (C) genes. Error bars represent the standard deviation of the values from at least three replicate assays. Different lowercase letters (a–c) denote significant differences (*P < *0.05) between treatment groups according to Tukey's test.

To further explore the potential roles of milRNAs in *S*. *vaninii*, the expression profiles of four *Svrdrps*, two *Svdicers*, and four *Svagos* in solid PDA (20 days), liquid PDS (10 days), and fruiting bodies (3 years old) were assessed with qPCR. The results showed that all 10 genes were actively transcribed at all investigated developmental stages (Fig. 2). In particular, the expression trends of all four *Svrdrps* were highly consistent in each stage and showed decreased expression in solid PDA samples compared with that in both liquid PDS and fruiting body samples (Fig. 2A). The highest gene expression levels of the two *Svdicers* were observed in the fruiting body, and *Svdicer-2* expression decreased from the liquid PDS sample to the solid PDA sample, while *Svdicer-1* from both samples showed no significant difference in expression (Fig. 2B). *Svago-1* and *Svago-3* showed decreased expression in the solid PDA sample compared with that in both the liquid PDS and fruiting body samples, while *Svago-2* and *Svago-4* revealed the lowest expression in the fruiting body and liquid PDS samples, respectively (Fig. 2C).

### Silencing of AGO, Dicer, and RdRP genes with dsRNA uptake.

**FIG 3:**
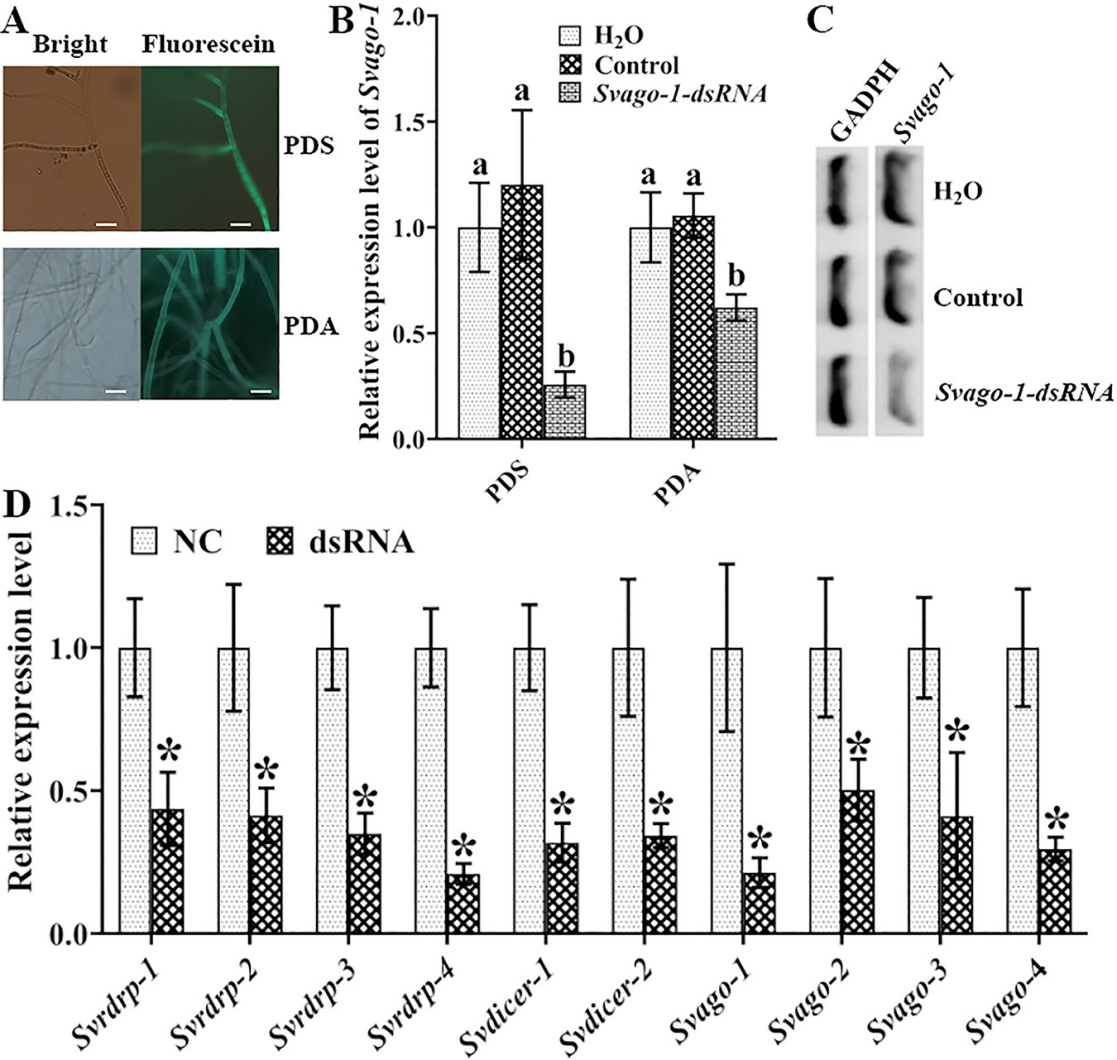
Silencing of AGO, Dicer and RdRP genes with dsRNA uptake. Examination of dsRNA uptake efficiency in *S. vaninii* from solid PDA and liquid PDS medium with fluorescein-labeled *Svago-dsRNA* by confocal microscopy laser scanner (A) and qPCR (B). The silencing efficiency of *Svago-1* was confirmed by using semiquantitative RT–PCR (C). qPCR analysis of RdRP, Dicer, and AGO gene expression levels in different dsRNA-treated strains (D). Scale bars = 5 μm; different lowercase letters (–b) and asterisks marked on the bars in each graph denote significant differences (*P < *0.05) between treatment groups according to Tukey's test. Error bars represent the standard deviation of the values from six replicate assays.

To determine whether dsRNA was taken up by *S*. *vaninii*, the efficiency of dsRNA uptake was tested with fluorescein-labeled *Svago-1* dsRNA (*Svago-1-dsRNA*). Mycelium from both solid PDA and liquid PDS medium were treated with fluorescein-labeled *Svago-1-dsRNA* to determine the fungal dsRNA uptake efficiency at different stages. Fluorescence signals were observed after the removal of labeled dsRNA from the fungal cells, and the results showed that mycelia from both solid PDA and liquid PDS medium showed very efficient dsRNA uptake (Fig. 3A). We also found that the expression levels of *Svago-1* and *Svago-1* were significantly downregulated after treatment with *Svago-1* dsRNA (Fig. 3B). Remarkably, dsRNA-targeted silencing was more efficiently taken up in liquid PDS than in solid PDA; thus, the fungus cultured in liquid PDS medium was used for gene silencing with dsRNA uptake in the following study. Moreover, the silencing efficiency of *Svago-1* was confirmed by using semiquantitative RT–PCR (Fig. 3C).

Combining the above results, silencing of the AGO, Dicer and RdRP genes was performed with dsRNA uptake. qPCR results showed that all genes were found to be significantly downregulated (2- to 5-fold decrease in transcript levels) in the dsRNA-treated fungus (Fig. 3D). Thus, the application of dsRNA uptake to silence the targeted genes could be an effective strategy to explore gene function in *S*. *vaninii*.

### Mycelial biomass and bioactive compound production from different gene-silenced fungi.

**FIG 4:**
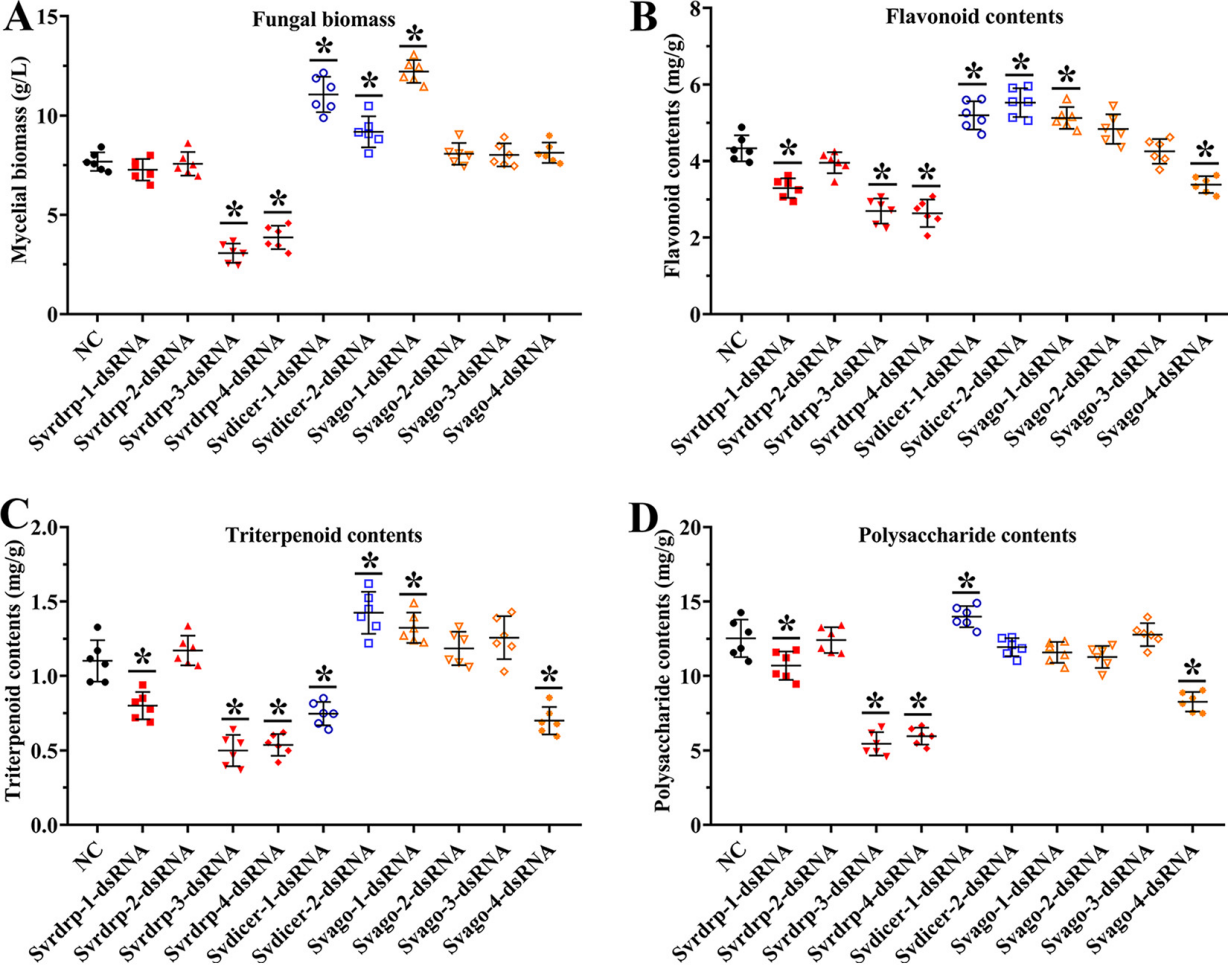
Mycelial biomass and bioactive compound yields from *S. vaninii* treated with gene-targeting dsRNAs. (A) Fungal biomass determined by measuring the mycelial dry weight. Yields of flavonoids (B), triterpenoids (C), and polysaccharides (D) were detected in different strains. The strain treated with *gfp-dsRNA* was used as the negative control (NC). Error bars represent the standard deviation of the values from six replicate assays. The asterisks marked on the bars in each graph denote significant differences (*P < *0.05).

To further explore the possible roles of the AGO, Dicer and RdRP genes in the production of bioactive compounds, yields of flavonoids, triterpenoids, and polysaccharides were detected. First, we determined the fungal biomass by measuring the mycelial dry weight. Compared with NC (gfp-dsRNA treatment), Svrdrp-3-dsRNA and Svrdrp-4-dsRNA showed considerable decreases, whereas Svdicer-1-dsRNA, Svdicer-2-dsRNA, and Svago-1-dsRNA revealed significant increases in mycelial biomass (Fig. 4A). Flavonoid production of Svdicer-1-dsRNA, Svdicer-2-dsRNA, and Svago-1-dsRNA was significantly higher than that in NC (Fig. 4B). However, compared with NC, a reduction was observed in flavonoid production in *Svrdrp-1*-, *Svrdrp-3*-, *Svrdrp-4*-, or *Svago-4*-silenced mycelium. For triterpenoid production, Svrdrp-1-dsRNA, Svrdrp-3-dsRNA, Svrdrp-4-dsRNA, Svdicer-1-dsRNA, and Svago-4-dsRNA were weaker but stronger in Svdicer-2-dsRNA and Svago-1-dsRNA than NC (Fig. 4C). Polysaccharide contents increased significantly by silencing *Svdicer-1* but decreased significantly after *Svrdrp-1*, *Svrdrp-3*, *Svrdrp-4*, or *Svago-4* was silenced in *S*. *vaninii* (Fig. 4D). Overall, *Svrdrp-3*, *Svrdrp-4*, *Svdicer-1*, and *Svdicer-2* were critical for mycelial biomass and the production of the three bioactive compounds.

### Analysis of milRNA characteristics in *S*. *vaninii*.

**FIG 5:**
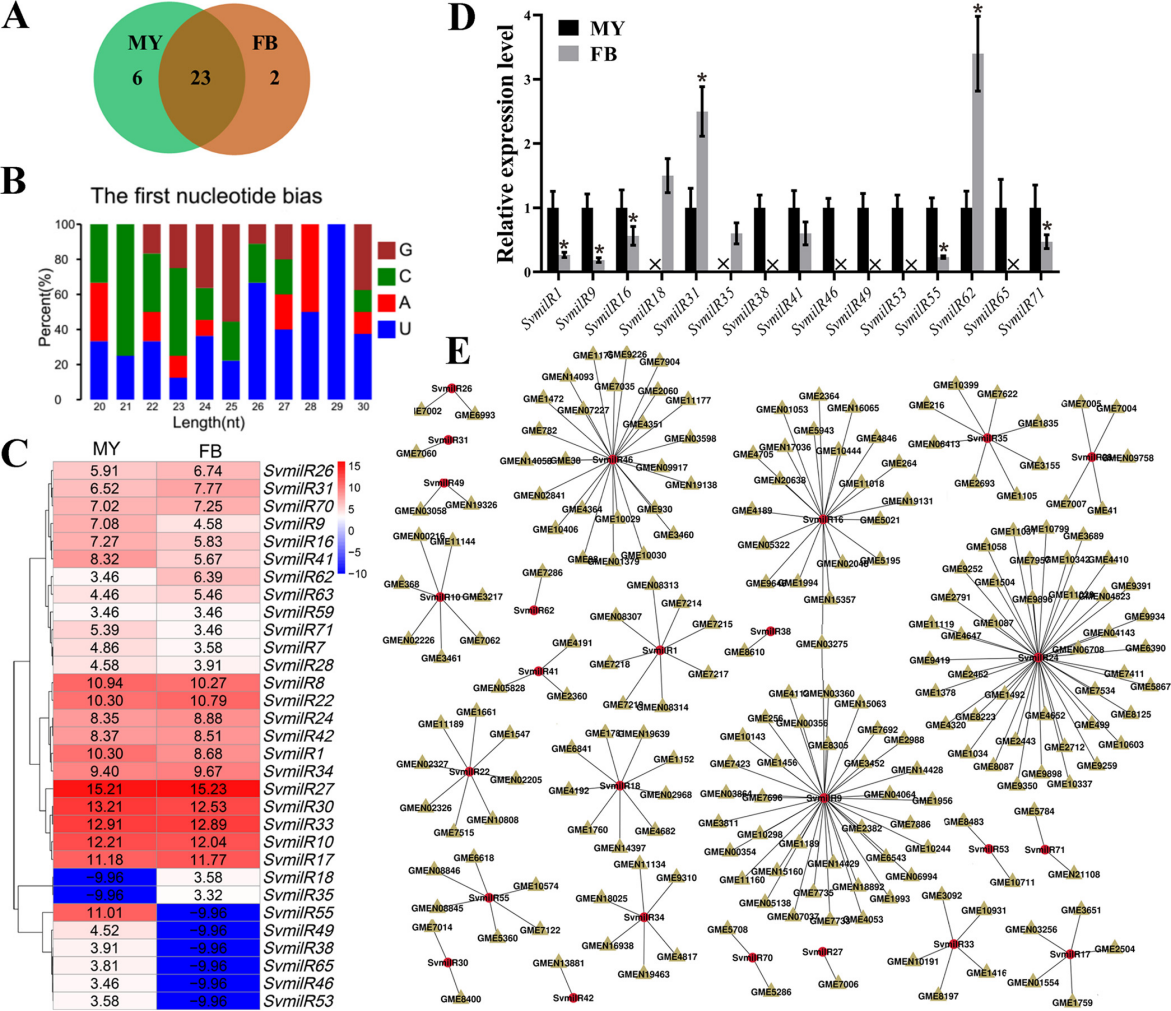
Characteristics and expression of milRNAs in *S. vaninii*. (A) The Venn diagram illustrates the number of milRNAs from liquid PDS mycelium (MY) and fruiting body (FB). (B) Analysis of nucleotide biases of milRNAs. (C) Heat-map diagram analysis of milRNA expression in MY and FB. (D) Validation of differentially expressed milRNAs from small RNA-Seq results with qPCR. (E) Network analysis of milRNAs and these target genes. The asterisks marked on the bars in each graph denote significant differences (*P < *0.05).

To investigate the milRNA characteristics, we analyzed the milRNAs generated in *S*. *vaninii* from liquid PDS medium-cultured fungus (MY) and fruiting body (FB). A total of 31 milRNAs were obtained, and six milRNAs were expressed exclusively in MY, whereas twenty-three were expressed in both MY and FB (Fig. 5A and Table S1). The first base at the 5′ terminus of milRNAs 21 to 23 bp in length had a strong preference for “C,” in contrast to milRNAs with lengths of 24 to 29 bp (Fig. 5B). All milRNA abundance was normalized with TPM normalization, and an absolute value of log_2_ ratio ≥ 1 (*P < *0.05) was used as the threshold to determine significant differences in milRNA expression. A total of 15 milRNAs were found to have significantly different expression between FB and MY: compared with their expression in MY, four milRNAs were upregulated while the remainder were downregulated in FB (Fig. 5C). To validate the high-sequencing data, we analyzed the expression profiles of all differentially expressed milRNAs with qPCR. The expression profiles of all 15 milRNAs were consistent with the sequencing data and showed significantly differential expression between FB and MY (Fig. 5D).

To understand the biological functions triggered by milRNAs, the target genes of these milRNAs were identified. A total of 213 unique coding genes were predicted to be targets of these milRNAs, and a total of 212 milRNA-target pairs were identified (Fig. 5E and Table S2). Moreover, analysis of interaction networks suggested that most of these milRNAs regulated several genes, and some milRNAs, such as *SvmilR9*, *SvmilR16*, *SvmilR24*, and *SvmilR46*, had connections with more than 20 genes.

### RdRP- and Dicer-dependent biogenesis of milRNAs involved in mycelial biomass and bioactive compound production.

**FIG 6:**
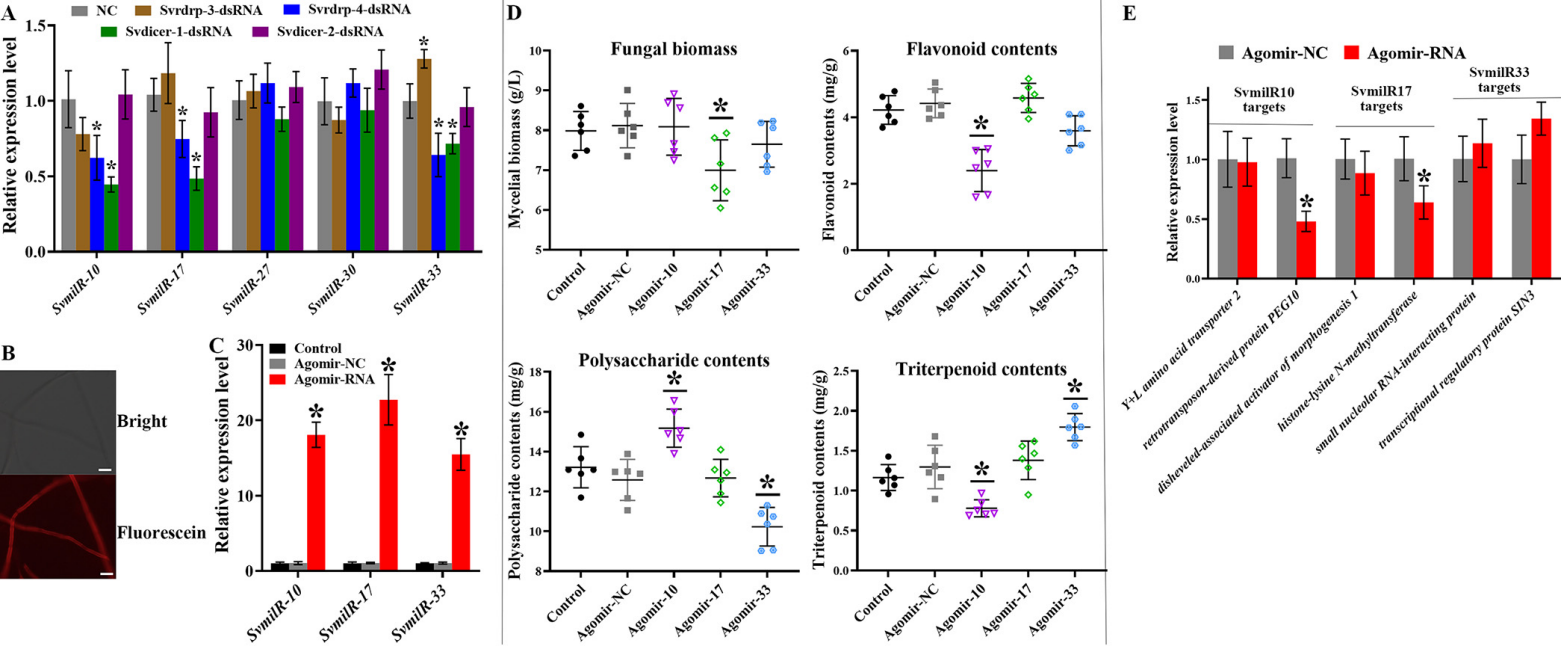
Roles of SVRDRP-4- and SVDICER-1-dependent milRNAs in mycelial biomass and bioactive compound yields. (A) qPCR analysis of milRNA expression from *S. vaninii* treated with gene-targeting dsRNAs. (B) sRNA uptake efficiency in *S. vaninii* from liquid PDS medium with Cy3-labeled agomir miRNA by confocal microscopy laser scanner. (C) milRNA expression analysis from different agomir miRNA-treated strains. (D) Mycelial biomass and bioactive compound yields from *S. vaninii* with overexpressed milRNAs. (E) qPCR analysis of the expression profiles of potential milRNA corresponding targets.

As in our study above, *Svrdrp-3*, *Svrdrp-4*, *Svdicer-1*, and *Svdicer-2* were critical for mycelial biomass and the production of the three bioactive compounds. To identify these gene-dependent milRNAs, the expression characteristics of five milRNAs with high abundance (more than 100 counts in both FB and MY) were examined from these gene-silenced fungi. Silencing *Svrdrp-4* or *Svdicer-1* resulted in a reduction in the expression of *SvmilR10*, *SvmilR17*, and *SvmilR33*, whereas *SvmilR33* showed increased expression of Svrdrp-3-dsRNA compared with that in NC (Fig. 6A). Thus, *SvmilR10*, *SvmilR17* and *SvmilR33* were regarded as *Svrdrp-4*- and *Svdicer-1*-dependent milRNAs.

To investigate whether synthetic milRNA was taken up by *S*. *vaninii*, Cy3-labeled miRNA was added to the liquid PDS culture of mycelium. Fluorescence signals were clearly observed at 48 h (Fig. 6B). Most importantly, the milRNA expression levels of *SvmilR10*, *SvmilR17* and *SvmilR33* were considerably increased (more than 15-fold increase in transcript levels) in *S*. *vaninii* treated with different agomir miRNAs (Fig. 6C). Thus, *S*. *vaninii* took up external miRNAs efficiently.

Next, we investigated the mycelial biomass and bioactive compound production from these agomir miRNA-treated fungi. Compared with the control (water treatment) and negative-control (NC) treatment, agomir-17 showed a significant reduction in fungal biomass. The yields of both flavonoids and polysaccharides were increased, while the production of triterpenoids was decreased in the fungi treated with agomir-10 (Fig. 6D). Overexpressed *SvmilR33* in the fungus showed the opposite change for the triterpenoid and polysaccharide contents, in which polysaccharide yield was decreased while triterpenoid yield was increased. Subsequently, the expression levels of predicted targets of *SvmilR10*, *SvmilR17* and *SvmilR33* were studied in agomir miRNA-treated fungi. The results showed that the gene encoding the retrotransposon-derived protein PEG10 was significantly decreased in agomir-10-treated fungus, and the expression level of the histone-lysine N-methyltransferase gene was downregulated in the fungus after agomir-10 treatment (Fig. 6E). These results indicate a role for *Svrdrp-4*- and *Svdicer-1*-dependent milRNAs in the regulation of mycelial biomass and bioactive compound production by gene silencing mediation.

## DISCUSSION

In fungi, the RdRP family generates dsRNA from certain single-stranded RNAs or from the target mRNA, activating or amplifying the silencing response, respectively. Different RDRP proteins are involved in diverse functional mechanisms; for example, RdRP1 is essential for the generation of antisense RNA from transgene transcripts, while RdRP2 amplifies the silencing signal in *Mucor lusitanicus* (27). In this study, four RdRPs were identified from *S. vaninii* and *S. baumii*, which was more than most filamentous fungi. Fungi exhibit diversity in the number of RdRPs, suggesting that milRNA-produced mechanisms are distinct among different fungi (28, 29). In addition, RdRP-3, RdRP-4, AGO-2, AGO-3, and AGO-4 from *S*. *vaninii* and *S*. *baumii* shared low similarity with the other fungal RdRPs and AGOs, suggesting that *Sanghuangporus* species may possess specific RNAi mechanisms.

Recently, impressive numbers of studies on SIGS indicated that RNAi is an effective control strategy against fungal pathogens and confirmed that fungi could take up environmental RNA (dsRNA and sRNA), which can then silence fungal genes through environmental RNA interference (16, 30). Undoubtedly, SIGS provides new strategies to study the gene function of some fungi that lack efficient gene manipulation systems. A recent study showed that *Sanghuangporus* species were binuclear fungi, which was limited for functional gene analysis with homologous recombination by DNA-mediated transformation and CRISPR/Cas9-based gene editing (9). Thus, RNA uptake efficiency was examined in *S*. *vaninii* because SIGS was dependent on the efficiency of fungal RNA uptake (31). We observed efficient dsRNA uptake in *S*. *vaninii* from both solid PDA and liquid PDS medium, but the silencing efficiency of fungus from PDA was less efficient than that from PDS, suggesting that differences in culture environment and cell types could affect fungal RNA uptake efficiency, which was consistent with an earlier report (16). In Fusarium graminearum, exogenous application of dsRNA triggering SIGS of *CYP51* was more efficient than host-induced gene silencing, suggesting that fungi seem to respond more efficiently to dsRNA than to siRNA (32). In this study, although milRNA abundances increased more than 15-fold, only weak characteristic changes occurred in the fungus, indicating low gene silencing efficiency by sRNA uptake. Therefore, exogenous application of dsRNA is a powerful genetic tool used for gene silencing. In fact, other gene editing technologies should be further explored in the fungus because of the time-consuming, high-cost, and low-efficiency stability of SIGS-based functional gene analysis.

In fungi, RdRP produces dsRNA molecules from single-stranded RNA precursors, and then Dicer cleaves the dsRNA and generates sRNAs (33). Fungal biomass and all three bioactive compound yields changed significantly in the *S*. *vaninii* treated with *Svrdrp-3*-, *Svrdrp-4*-, *Svdicer-1*-, or *Svdicer-2*-targeted dsRNAs, suggesting that *Svrdrp-3*-, *Svrdrp-4*-, *Svdicer-1*-, or *Svdicer-2*-regulated milRNAs were involved in fungal development and secondary metabolite production. The AGO protein forms a complex (RISC, RNA-induced silencing complex) to block gene transcription by targeting complementary specific miRNA sequences as a guide (34). MiRNAs were loaded into AGO4 in the plant cytoplasm and then mediated DNA methylation in the nucleus to target gene translational repression (35). In this study, silencing *Svago-4* resulted in a decrease in the yield of all three bioactive compounds in *S*. *vaninii*, indicating that miRNAs regulated the production of these compounds within SVAGO-4 to form RISC.

In *G. lucidum*, F. graminearum and *C. militaris*, 134, 143 and 38milRNAs were expressed in the sexual developmental stage, and only 92, 49, and 19 milRNAs were obtained from the asexual development stage (22, 29, 36). A total of 31 milRNAs were obtained in *S*. *vaninii*, twenty-nine milRNAs were detected in the asexual stage (MY), and 25 milRNAs were expressed in the sexual developmental stage (FB), suggesting that milRNAs were needed for asexual development rather than for sexual development in *S*. *vaninii*, which is contrary to these fungi. Although thirty-one milRNAs were obtained in *S*. *vaninii*, we have not been able to find any of the milRNAs to be conserved, which is consistent with *G. lucidum*, F. graminearum and *C. militaris*. Species-specific miRNAs are considered to be miRNAs that have evolved recently; thus, milRNAs may have evolved independently and recently in fungi (37).

Various studies have shown the important aspect of miRNAs in regulating secondary metabolite biosynthesis in different plant species (35). For example, miR828a and miR396b target anthocyanin regulatory protein-C1 and kaempferol 3-O-beta-d-galactosyltransferase genes in *Rauvolfia serpentine* (35, 38). In *Arabidopsis*, miR5021 targets genes encoding the GcpE protein and terpene cyclase involved in terpenoid biosynthesis, miR858a targets genes encoding the R2R3-MYB transcription factor involved in regulating flavonoid biosynthesis, and miR156 regulates anthocyanin accumulation by targeting the *SPL9* gene (39–41). Here, we found that SVRDRP-3, SVRDRP-4, SVDICER-1, and SVDICER-2 were critical for bioactive compound production, and *SvmilR10*, *SvmilR17,* and *SvmilR33* were regarded as *Svrdrp-4*- and *Svdicer-1*-dependent milRNAs. In the *SvmilR10*-overexpressing strain, flavonoid and polysaccharide contents were increased but triterpenoid production was decreased. One SvmilR10 target, the retrotransposon-derived protein PEG10 gene, was critical for normal cell homeostasis and downregulated in the strain after Agomir-10 uptake, suggesting that *SvmilR10* could control bioactive compound production with gene regulation (42, 43). Furthermore, overexpression of *SvmilR33* in the fungus resulted in decreased polysaccharide contents but increased triterpenoid yield, suggesting that different molecular mechanisms were used by milRNAs while producing different bioactive compounds.

In summary, core proteins of the miRNA-based silencing mechanism (RdRPs, Dicers and AGOs) and milRNAs were analyzed and characterized from the medicinal fungus *S. vaninii*. Importantly, milRNA roles in fungal biomass and bioactive compound production were analyzed by gene silencing with external dsRNA and sRNA uptake. These results suggested that milRNAs may be involved in the biosynthesis of bioactive compounds in *S. vaninii*.

## MATERIALS AND METHODS

### Fungal strain and culture conditions.

The fungus used in this study was *S. vaninii Kangneng*, which has been used for large-scale industrial production in Jiangsu KONEN Biological Engineering Co. Ltd. (Anhui, China) (8, 26). PDA plates (20% potato, 2% dextrose, and 1.5% agar, wt/vol) were used for the fungal culture at 28°C in the dark. A total of 250 mL flasks containing 100 mL of liquid PDS (20% potato, 2% mulberry tree sawdust) medium inoculated with the mycelia were used for the collection of mycelia from liquid medium. For fruiting body production, 10-day-cultured mycelia from liquid medium were inoculated in short log sections as described in our previous study (8).

### Phylogenetic analyses of RdRPs, Dicers, and AGOs.

RdRPs, Dicers, and AGOs from *S. vaninii* (SV), *Sanghuangporus baumii* (SB); Neurospora crassa (NC); Aspergillus flavus (AF); and Arabidopsis thaliana (AT) were used for phylogenetic analysis to further determine these conserved RNAi proteins (29). The BLASTp algorithm, underpinned by the Pfam and CDD databases, was used for searches of conserved protein domains or motifs. The amino acid sequences of RdRP, Dicer, and AGO proteins from different organisms were obtained from GenBank according to our previous study (29). Alignment sequences were used for phylogenetic analysis by MEGA7 (http://www.megasoftware.net) using a neighbor-joining method.

### RNA isolation, quantitative PCR (qPCR) and stem–loop real-time PCR.

According to our previous study, the production of bioactive compounds from *S*. *vaninii* was different at different stages; in this study, milRNA functions in the biosynthesis of active compounds were explored. Thus, fungal samples grown at the stage with high bioactive compound production were collected with different cultivation methods (solid PDA, liquid PDS and fruiting bodies) (26). Different fungal samples collected from solid PDA (20 days), liquid PDS (10 days), and fruiting bodies (3 years old) were used for RNA extraction with TRIzol reagent (Invitrogen, Carlsbad, CA, USA) according to the manufacturer’s instructions. RNase-free DNase I was used for DNA genome removal, and the RNA concentration was evaluated. Then, cDNA was synthesized with a gDNA remover kit (Toyobo, Osaka, Japan) according to the manufacturer’s instructions. qPCR amplification was performed with a SYBR green kit (TaKaRa) and Bio-Rad CFX96TM system (Hercules, CA, United States). Transcripts of different protein-coding genes were normalized to the control gene. The stem–loop real-time PCR method was performed to quantitate milRNA expression. In brief, a stem–loop RT primer was used to reverse-transcribe mature milRNAs to complementary DNAs (cDNAs). The 20 μL reverse-transcription reactions contained 500 ng of total RNA, 50 nM each individual stem–loop RT primer, 0.5 mM dNTP (TaKaRa, Dalian, China), 10 U M-MLV reverse transcriptase (TaKaRa, Dalian, China), and 4 U RNase inhibitor. qPCR amplification was performed as mentioned above. *Glyceraldehyde 3-phosphate dehydrogenase* (GAPDH) was used as the control. The relative gene expression levels were calculated with the 2^−ΔΔCt^ method. All experiments were performed with six replicates. Details of the primers used in this study are given in Table S3.

### *In vitro* synthesis of dsRNA and agomir milRNA.

To silence different core components of the RNAi machinery in *S*. *vaninii*, dsRNAs targeting *RNA-dependent RNA polymerase*s, *dicers*, and *argonautes* were synthesized with T7 RNA polymerase (T7 RiboMAX Express RNAi system kit, Promega, Madison, WI, USA) based on established protocols (44). Briefly, ~300 bp DNA fragments, introduced at both the 5′ and 3′ ends with the T7 promoter sequence, were produced via PCR (PCR) from cDNA. The DNA fragments were used for *in vitro* transcription after confirmation by DNA sequencing, and then the final dsRNA was dissolved in nuclease-free water. The green fluorescent protein (GFP) gene was used as the negative control. Fluorescein-labeled dsRNA was synthesized with a Fluorescein RNA Labeling Mix kit following the manufacturer’s instructions (Sigma, St. Louis, MO, USA) as previously described (45). Agomir miRNA, chemically modified and cholesterylated, was a stable miRNA mimic and synthesized by Shanghai GenePharma (GenePharma, Shanghai, China).

### Fungal uptake of dsRNA or agomir miRNA *in vitro*.

Conidia from the fungus grown on PDA medium were counted and diluted to 5 × 10^6^ spores/mL. Fifty μL of conidial suspension was transferred into 20 mL of liquid PDS or solid PDA medium cultured at 28°C in the dark for 10 days. Then, 100 μL of dsRNA (100 ng/μL) or agomir miRNA (50 ng/μL) was added to the liquid PDS medium or evenly spread over the surface of the solid PDA medium by brushing lightly with a soft brush. Synthesized dsRNA targeting *gfp* (*gfp-dsRNA*) was used as the negative control (NC). Before confocal microscopy and qPCR examination, dsRNA or agomir miRNA outside the fungal cells was removed by washing four times with sterilized water. Fungal mycelium was added to external *gfp-dsRNA* or agomir miRNA (micrON agomir NC number 24), and the mixtures were immediately used for dsRNA or agomir miRNA removal. Then, total RNA was extracted for qPCR to ensure clean external RNA removal.

For confocal microscopy examination, fungal mycelium was collected from PDS or PDA medium after 48 h of culture with the addition of fluorescent dsRNA or Cy3-labeled agomir miRNA. The fluorescent signal was analyzed using a confocal microscope (Zeiss LSM 880, Oberkochen, Germany).

### Determination of active compounds.

The yields of the active ingredients (polysaccharides, flavonoids, and triterpenoid) among fungi treated with dsRNA or agomir miRNA were determined as described in our previous report (26). In brief, samples were dried and ground into powder, and 50 mg of powder was used for the extraction and determination of polysaccharide, flavonoid, and triterpenoid contents by measuring the absorbance at 620 nm, 384 nm, and 551 nm, respectively.

### sRNA library construction and sequencing.

The mycelium (MY: harvested after 10 days of growth in the PDS medium) and fruiting bodies (FB: 3 years old), currently widely used methods for the *Sanghuangporus* collection, of *S*. *vaninii* were used to construct small RNA and cDNA libraries. For fungal sRNA library construction, fresh fruiting bodies were pulverized to powder as previously described (26). Samples were ground into a powder using liquid nitrogen, and RNAiso Plus reagent (TaKaRa, Shiga, Japan) was used for total RNA extraction. A NanoDrop ND-2000 spectrophotometer (NanoDrop Technologies, Wilmington, DE, United States) and an Agilent 2100 Bioanalyzer (Agilent, United States) were used for RNA concentration evaluation after genomic DNA removal with RNase-free DNase I. To maximize target coverage, equal amounts of total RNA from each of the three replicates of samples were pooled for library construction. RNA (15 to 30 nt) was collected from total RNA on a 15% denaturing polyacrylamide gel, and then 5′ and 3′ of these RNAs were ligated to specific adaptors. cDNA libraries were sequenced with the Illumina HiSeq 2000 platform (BGI, Shenzhen, China) after reverse transcription.

Data analysis of small RNAs was performed as described in a previous study (29). Briefly, clean reads were obtained from raw reads by filtering out poor-quality reads after adaptor sequence removal. Adaptor-trimmed unique sequences were aligned to the *S. vaninii* genome (https://www.ncbi.nlm.nih.gov/nuccore/VVIT00000000.1/). Known noncoding RNAs from mapped reads, such as tRNA, snoRNA, and rRNA, were removed, and the remaining reads were aligned with mature miRNA sequences in the miRbase database to identify known milRNAs. According to the characteristics of the precursor of miRNA (a typical hairpin structure), the mideep 2 (https://www.mdc-berlin.de/content/mirdeep2-documentation) method was used to predict new miRNAs, and the secondary structure was successfully predicted. The abundance of milRNAs was normalized according to transcripts per million (TPM) normalization, and milRNA prediction was performed with PsRobot (https://tools4mirs.org/software/target_prediction/psrobot/) and TargetFinder (http://plantgrn.noble.org/psRNATarget/) according to plantlike target interactions and their targets with default parameters (35, 46).

### Statistical analysis.

Data were analyzed by one-way analysis of variance (ANOVA) and Tukey’s HSD test. SPSS v23.0 software for Windows (SPSS Inc., Chicago, IL, USA) was used for statistical analysis. All results are expressed as the mean ± standard error of the mean (SD). *P < *0.05 was considered significant.

### Data availability.

Data are available in a public, open access repository. All sequence data from this study has been submitted to Sequence Read Archive (https://www.ncbi.nlm.nih.gov/sra) and can be accessed with accessions PRJNA798153, SRR17649062, and SRR17649063.

## References

[1] Panda AK, Swain KC. 2011. Traditional uses and medicinal potential of *Cordyceps sinensis* of Sikkim. J Ayurveda Integr Med 2:9–13. doi:10.4103/0975-9476.78183. 21731381 PMC3121254

[2] Chen S, Xu J, Liu C, Zhu Y, Nelson DR, Zhou S, Li C, Wang L, Guo X, Sun Y, Luo H, Li Y, Song J, Henrissat B, Levasseur A, Qian J, Li J, Luo X, Shi L, He L, Xiang L, Xu X, Niu Y, Li Q, Han MV, Yan H, Zhang J, Chen H, Lv A, Wang Z, Liu M, Schwartz DC, Sun C. 2012. Genome sequence of the model medicinal mushroom *Ganoderma lucidum*. Nat Commun 3:913. doi:10.1038/ncomms1923. 22735441 PMC3621433

[3] Lu HY, Lou HH, Hu JJ, Liu ZJ, Chen QH. 2020. Macrofungi: a review of cultivation strategies, bioactivity, and application of mushrooms. Compr Rev Food Sci Food Saf 19:2333–2356. doi:10.1111/1541-4337.12602. 33336985

[4] Bandara AR, Rapior S, Bhat DJ, Kakumyan P, Chamyuang S, Xu JC, Hyde KD. 2015. *Polyporus umbellatus*, an edible-medicinal cultivated mushroom with multiple developed health-care products as food, medicine and cosmetics: a review. Cryptogamie Mycol 36:3–42. doi:10.7872/crym.v36.iss1.2015.3.

[5] Zhou L-W, Ghobad-Nejhad M, Tian X-M, Wang Y-F, Wu F. 2020. Current status of ‘Sanghuang’ as a group of medicinal mushrooms and their perspective in industry development. Food Rev Int 1–19. doi:10.1080/87559129.2020.1858859.

[6] Jiang J-H, Wu S-H, Zhou L-W. 2021. The first whole genome sequencing of *Sanghuangporus sanghuang* provides insights into its medicinal application and evolution. JoF 7:787. doi:10.3390/jof7100787. 34682209 PMC8537844

[7] Zhu L, Song J, Zhou JL, Si J, Cui BK. 2019. Species diversity, phylogeny, divergence time, and biogeography of the genus *Sanghuangporus* (Basidiomycota). Front Microbiol 10:812. doi:10.3389/fmicb.2019.00812. 31057518 PMC6478708

[8] Shao Y, Guo H, Zhang J, Liu H, Wang K, Zuo S, Xu P, Xia Z, Zhou Q, Zhang H, Wang X, Chen A, Wang Y. 2019. The Genome of the medicinal macrofungus sanghuang provides insights into the synthesis of diverse secondary metabolites. Front Microbiol 10:3035. doi:10.3389/fmicb.2019.03035. 31993039 PMC6971051

[9] Huo J, Zhong S, Du X, Cao Y, Wang W, Sun Y, Tian Y, Zhu J, Chen J, Xuan L, Wu C, Li Y. 2020. Whole-genome sequence of *Phellinus gilvus* (mulberry Sanghuang) reveals its unique medicinal values. J Adv Res 24:325–335. doi:10.1016/j.jare.2020.04.011. 32455007 PMC7235939

[10] Chung C-L, Lee TJ, Akiba M, Lee H-H, Kuo T-H, Liu D, Ke H-M, Yokoi T, Roa MB, Lu M-YJ, Chang Y-Y, Ann P-J, Tsai J-N, Chen C-Y, Tzean S-S, Ota Y, Hattori T, Sahashi N, Liou R-F, Kikuchi T, Tsai IJ. 2017. Comparative and population genomic landscape of *Phellinus noxius*: a hypervariable fungus causing root rot in trees. Mol Ecol 26:6301–6316. doi:10.1111/mec.14359. 28926153

[11] Oghenekaro AO, Kovalchuk A, Raffaello T, Camarero S, Gressler M, Henrissat B, Lee J, Liu M, Martínez AT, Miettinen O, Mihaltcheva S, Pangilinan J, Ren F, Riley R, Ruiz-Dueñas FJ, Serrano A, Thon MR, Wen Z, Zeng Z, Barry K, Grigoriev IV, Martin F, Asiegbu FO. 2020. Genome sequencing of *Rigidoporus microporus* provides insights on genes important for wood decay, latex tolerance and interspecific fungal interactions. Sci Rep 10:1–15. doi:10.1038/s41598-020-62150-4. 32251355 PMC7089950

[12] Premsrirut PK, Dow LE, Kim SY, Camiolo M, Malone CD, Miething C, Scuoppo C, Zuber J, Dickins RA, Kogan SC, Shroyer KR, Sordella R, Hannon GJ, Lowe SW. 2011. A rapid and scalable system for studying gene function in mice using conditional RNA interference. Cell 145:145–158. doi:10.1016/j.cell.2011.03.012. 21458673 PMC3244080

[13] Pampolini F, Rodrigues TB, Leelesh RS, Kawashima T, Rieske LK. 2020. Confocal microscopy provides visual evidence and confirms the feasibility of dsRNA delivery to emerald ash borer through plant tissues. J Pest Sci 93:1143–1153. doi:10.1007/s10340-020-01230-w.

[14] Wang M, Weiberg A, Lin FM, Thomma BPHJ, Huang HD, Jin HL. 2016. Bidirectional cross-kingdom RNAi and fungal uptake of external RNAs confer plant protection. Nat Plants 2:1–10. 10.1038/nplants.2016.151 PMC5040644 27643635

[15] Billmyre RB, Calo S, Feretzaki M, Wang X, Heitman J. 2013. RNAi function, diversity, and loss in the fungal kingdom. Chromosome Res 21:561–572. doi:10.1007/s10577-013-9388-2. 24173579 PMC3874831

[16] Qiao L, Lan C, Capriotti L, Ah-Fong A, Sanchez N, Hamby R, Heller J, Zhao H, Glass NL, Judelson HS, Mezzetti B, Niu D, Jin H. 2021. Spray-induced gene silencing for disease control is dependent on the efficiency of pathogen RNA uptake. Plant Biotechnol J 19:1756–1768. doi:10.1111/pbi.13589. 33774895 PMC8428832

[17] Wytinck N, Manchur CL, Li VH, Whyard S, Belmonte MF. 2020. dsRNA uptake in plant pests and pathogens- insights into rnai-based insect and fungal control technology. Plants 9:1780. doi:10.3390/plants9121780. 33339102 PMC7765514

[18] Lee HC, Li L, Gu W, Xue Z, Crosthwaite SK, Pertsemlidis A, Lewis ZA, Freitag M, Selker EU, Mello CC, Liu Y. 2010. Diverse pathways generate microRNA-like RNAs and Dicer-independent small interfering RNAs in fungi. Mol Cell 38:803–814. doi:10.1016/j.molcel.2010.04.005. 20417140 PMC2902691

[19] Guo MW, Yang P, Zhang JB, Liu G, Yuan QS, He WJ, Nian JN, Yi SY, Huang T, Liao YC. 2019. Expression of microRNA-like RNA-2 (Fgmil-2) and bioH1 from a single transcript in *Fusarium graminearum* are inversely correlated to regulate biotin synthesis during vegetative growth and host infection. Mol Plant Pathol 20:1574–1581. doi:10.1111/mpp.12859. 31385410 PMC6804420

[20] Torres-Martínez S, Ruiz-Vázquez RM. 2017. The RNAi universe in fungi: a varied landscape of small rnas and biological functions. Annu Rev Microbiol 71:371–391. doi:10.1146/annurev-micro-090816-093352. 28657888

[21] Mu DS, Li CY, Shi L, Zhang XC, Ren A, Zhao MW. 2015. Bioinformatic identification of potential micrornas and their targets in the lingzhi or reishi medicinal mushroom *Ganoderma lucidum* (Higher Basidiomycetes). Int J Med Mushrooms 17:783–797. doi:10.1615/intjmedmushrooms.v17.i8.80. 26559864

[22] Shao J, Wang L, Liu Y, Qi Q, Wang B, Lu S, Liu C. 2020. Identification of milRNAs and their target genes in *Ganoderma lucidum* by high-throughput sequencing and degradome analysis. Fungal Genet Biol 136:103313. doi:10.1016/j.fgb.2019.103313. 31751775

[23] Lau AYT, Xie YC, Cheung MK, Cheung PCK, Kwan HS. 2020. Genome-wide mRNA and miRNA analysis in the early stages of germ tube outgrowth in *Coprinopsis cinerea*. Fungal Genet Biol 142:103416. doi:10.1016/j.fgb.2020.103416. 32522620

[24] Lau AYT, Cheng X, Cheng CK, Nong W, Cheung MK, Chan RH, Hui JHL, Kwan HS. 2018. Discovery of microRNA-like RNAs during early fruiting body development in the model mushroom *Coprinopsis cinerea*. PLoS One 13:e0198234. doi:10.1371/journal.pone.0198234. 30231028 PMC6145500

[25] Gong M, Yang R, Wu Y, Li Y, Tan Q, Wang Y, Zhang J, Zhao Y, Wan J, Shang J, Bao D. 2020. Chilling stress triggers vvago1-mediated mirna-like rna biogenesis in *Volvariella volvacea*. Front Microbiol 11:523593. doi:10.3389/fmicb.2020.523593. 33042047 PMC7522536

[26] Zhou Q, Wang J, Jiang H, Wang G, Wang Y. 2021. Deep sequencing of the Sanghuangporus vaninii transcriptome reveals dynamic landscapes of candidate genes involved in the biosynthesis of active compounds. Arch Microbiol 203:2315–2324. doi:10.1007/s00203-021-02225-6. 33646337

[27] Lax C, Tahiri G, Patiño Medina A, Canovas Marquez JT, Perez Ruiz JA, Osorio Concepción M, Navarro E, Calo S. 2020. The evolutionary significance of RNAi in the fungal kingdom. Int J Mol Sci 21:9348. doi:10.3390/ijms21249348. 33302447 PMC7763443

[28] Yang JS, Lai EC. 2011. Alternative miRNA biogenesis pathways and the interpretation of core miRNA pathway mutants. Mol Cell 43:892–903. doi:10.1016/j.molcel.2011.07.024. 21925378 PMC3176435

[29] Shao Y, Tang J, Chen S, Wu Y, Wang K, Ma B, Zhou Q, Chen A, Wang Y. 2019. milR4 and milR16 mediated fruiting body development in the medicinal fungus *Cordyceps militaris*. Front Microbiol 10:83. doi:10.3389/fmicb.2019.00083. 30761116 PMC6362416

[30] Secic E, Kogel KH. 2021. Requirements for fungal uptake of dsRNA and gene silencing in RNAi-based crop protection strategies. Curr Opin Biotechnol 70:136–142. doi:10.1016/j.copbio.2021.04.001. 34000482

[31] Koch A, Wassenegger M. 2021. Host-induced gene silencing - mechanisms and applications. New Phytol 231:54–59. doi:10.1111/nph.17364. 33774815

[32] Koch A, Hofle L, Werner BT, Imani J, Schmidt A, Jelonek L, Kogel KH. 2019. SIGS vs HIGS: a study on the efficacy of two dsRNA delivery strategies to silence *Fusarium* FgCYP51 genes in infected host and non-host plants. Mol Plant Pathol 20:1636–1644. doi:10.1111/mpp.12866. 31603277 PMC6859480

[33] Nicolas FE, Garre V. 2016. RNA interference in fungi: retention and loss. Microbiol Spectr 4:4–6. doi:10.1128/microbiolspec.FUNK-0008-2016. 28087943

[34] Choi J, Kim KT, Jeon J, Wu JY, Song H, Asiegbu FO, Lee YH. 2014. funRNA: a fungi-centered genomics platform for genes encoding key components of RNAi. BMC Genomics 15:1–10. doi:10.1186/1471-2164-15-S9-S14. 25522231 PMC4290597

[35] Jeena GS, Singh N, Shukla RK. 2022. An insight into microRNA biogenesis and its regulatory role in plant secondary metabolism. Plant Cell Rep 41:1651–1671. doi:10.1007/s00299-022-02877-8. 35579713

[36] Zeng W, Wang J, Wang Y, Lin J, Fu Y, Xie J, Jiang D, Chen T, Liu H, Cheng J. 2018. Dicer-like proteins regulate sexual development via the biogenesis of *Perithecium*-specific microRNAs in a plant pathogenic fungus *Fusarium graminearum*. Front Microbiol 9:818. doi:10.3389/fmicb.2018.00818. 29755439 PMC5932338

[37] Allen E, Xie ZX, Gustafson AM, Sung GH, Spatafora JW, Carrington JC. 2004. Evolution of microRNA genes by inverted duplication of target gene sequences in *Arabidopsis thaliana*. Nat Genet 36:1282–1290. doi:10.1038/ng1478. 15565108

[38] Prakash P, Rajakani R, Gupta V. 2016. Transcriptome-wide identification of *Rauvolfia serpentina* microRNAs and prediction of their potential targets. Comput Biol Chem 61:62–74. doi:10.1016/j.compbiolchem.2015.12.002. 26815768

[39] Gou JY, Felippes FF, Liu CJ, Weigel D, Wang JW. 2011. Negative regulation of anthocyanin biosynthesis in *Arabidopsis* by a miR156-targeted SPL transcription factor. Plant Cell 23:1512–1522. doi:10.1105/tpc.111.084525. 21487097 PMC3101539

[40] Oudin A, Mahroug S, Courdavault V, Hervouet N, Zelwer C, Rodríguez-Concepcion M, St-Pierre B, Burlat V. 2007. Spatial distribution and hormonal regulation of gene products from methylerythritol phosphate and monoterpene-secoiridoid pathways in *Catharanthus roseus*. Plant Mol Biol 65:13–30. doi:10.1007/s11103-007-9190-7. 17611800

[41] Sharma D, Tiwari M, Pandey A, Bhatia C, Sharma A, Trivedi PK. 2016. MicroRNA858 is a potential regulator of phenylpropanoid pathway and plant development. Plant Physiol 171:944–959. doi:10.1104/pp.15.01831. 27208307 PMC4902582

[42] Ono R, Nakamura K, Inoue K, Naruse M, Usami T, Wakisaka-Saito N, Hino T, Suzuki-Migishima R, Ogonuki N, Miki H, Kohda T, Ogura A, Yokoyama M, Kaneko-Ishino T, Ishino F. 2006. Deletion of Peg10, an imprinted gene acquired from a retrotransposon, causes early embryonic lethality. Nat Genet 38:101–106. doi:10.1038/ng1699. 16341224

[43] Lux H, Flammann H, Hafner M, Lux A. 2010. Genetic and molecular analyses of PEG10 reveal new aspects of genomic organization, transcription and translation. PLoS One 5:e8686. doi:10.1371/journal.pone.0008686. 20084274 PMC2800197

[44] Wang Y, Zhou Q, Zhang H, Qin L, Huang B. 2021. Immunotranscriptome analysis of *Plutella xylostella* reveals differences in innate immune responses to low- and high-virulence *Beauveria bassiana* strain challenges. Pest Manag Sci 77:1070–1080. doi:10.1002/ps.6124. 33015931

[45] Hamby R, Wang M, Qiao L, Jin H. 2020. Synthesizing fluorescently labeled dsRNAs and sRNAs to visualize fungal RNA uptake, p 215–225. RNA Tagging. Humana, New York, NY. 10.1007/978-1-0716-0712-1_12 32710411

[46] Hoen PAC, Ariyurek Y, Thygesen HH, Vreugdenhil E, Vossen RHAM, de Menezes RX, Boer JM, van Ommen GJB, den Dunnen JT. 2008. Deep sequencing-based expression analysis shows major advances in robustness, resolution and inter-labportability over five microarray platforms. Nucleic Acids Res 36:e141. doi:10.1093/nar/gkn705. 18927111 PMC2588528

